# Effects of Tocotrienol and Lovastatin Combination on Osteoblast and Osteoclast Activity in Estrogen-Deficient Osteoporosis

**DOI:** 10.1155/2012/960742

**Published:** 2012-08-13

**Authors:** Saif Abdul-Majeed, Norazlina Mohamed, Ima-Nirwana Soelaiman

**Affiliations:** Department of Pharmacology, Faculty of Medicine, Universiti Kebangsaan Malaysia, Jalan Raja Muda Abdul Aziz, 50300 Kuala Lumpur, Malaysia

## Abstract

Statins are HMGCoA reductase inhibitors and had been demonstrated to stimulate bone formation in rodents after high oral doses. Observational studies on patients treated with oral statins were varied. Delta-tocotrienol had been found to stimulate the cleavage of HMGCoA reductase and inhibit its activity. Tocotrienols were found to have both catabolic and anabolic effects on bone in different animal models of osteoporosis. The current study aimed to ascertain the effects of delta–tocotrienol and lovastatin combination on biochemical and static bone histomorphometric parameters in a postmenopausal rat model at clinically tolerable doses. 48 Sprague Dawley female rats were randomly divided into 6 groups: (1) baseline control group; (2) sham-operated control group; (3) ovariectomised control group; (4) ovariectomised and 11 mg/kg lovastatin; (5) ovariectomised and 60 mg/kg delta-tocotrienol; (6) ovariectomised and 60 mg/kg delta-tocotrienol + 11 mg/kg lovastatin. These treatments were given daily via oral gavage for 8 weeks. Delta-tocotrienol plus lovastatin treatment significantly increased bone formation and reduced bone resorption compared to the other groups. Therefore, the combined treatment may have synergistic or additive effects and have the potential to be used as an antiosteoporotic agent in patients who are at risk of both osteoporosis and hypercholesterolemia, especially in postmenopausal women.

## 1. Introduction

Osteoporosis is known as a silent age-related disorder, and it is considered as a major public health problem. Patients with osteoporosis have decreased bone density and microarchitectural disruption of bone tissue, leading to skeletal fragility and fractures. Postmenopausal osteoporosis is the most common type associated with high bone turnover and is due to estrogen deficiency [1]. Current available therapies are effective in the prevention of bone loss by stabilizing the bone mass through inhibition of osteoclast activity, but they are not favored to treat established osteoporosis where there is a need to increase bone volume. The United States Food and Drug Administration approved parathyroid hormone (Teriparatide) in 2002 as the first bone anabolic agent that can reduce the risk of osteoporotic fractures and increase bone mineral density [2]. However, the use of parathyroid hormone is associated with some drawbacks such as daily injection, and the possibility of tumorigenesis [3]. The identification of a well-tolerated anabolic agent that can increase bone formation and restore bone strength would represent a major therapeutic breakthrough in the treatment of any form of bone loss.

3-hydroxy-3-methylglutaryl coenzyme A (HMGCoA) reductase catalyzes the conversion of HMGCoA to mevalonic acid. Statins are competitive and reversible inhibitors of HMGCoA reductase. They are safely used as cholesterol-lowering agents and have pleiotropic actions in various systems such as the cardiovascular system, immune system, and nervous system [4]. Lovastatin is a prodrug and is converted to the active open-ring acid from its lactone by esterases. Lovastatin was the first compound identified as a promising bone anabolic agent after examining about 30,000 compounds [5]. Statins act as an anabolic agent by promoting bone formation *in vitro* and also *in vivo* in rodents after high oral doses [5–11]. Several observational clinical studies on patients treated with oral statins showed varying results. Some had suggested that oral statins minimize the risk of fractures and increase bone mineral density [12–17], while others reported that they had no effects on bone [18–23]. Several clinical studies that compared bone biochemical markers between statin-treated patients and control populations have had varying outcomes [24–26]. However, these findings as a whole suggested that the oral statins do not have sufficient anabolic effects *in vivo* when given in cholesterol lowering doses. Therefore, high doses of statins are needed to protect the bone and induce bone formation *in vivo*. However, high doses of statins had been associated with myotoxicity and hepatotoxicity [27–29].

**Figure 1:**
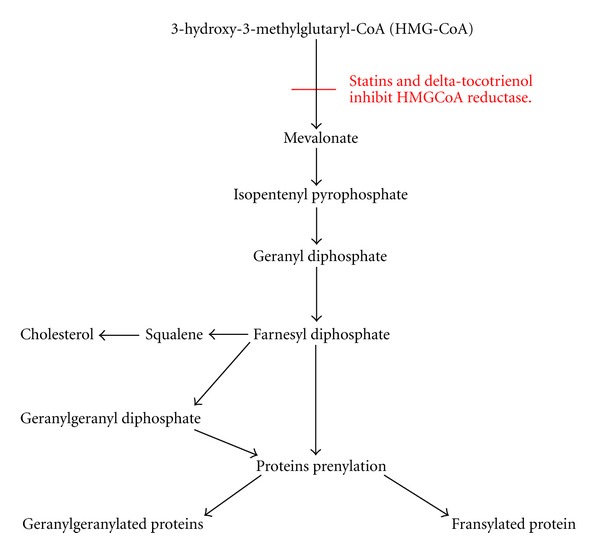
Mechanism of action of lovastatin and delta-tocotrienol on mevalonate pathway.

Tocotrienols and tocopherols are members of the vitamin E family. They are further subdivided into alpha, beta, gamma, and delta isomers. All the vitamin E isomers have antioxidant properties. In addition, tocotrienols have anticancer, neuroprotective, antiplatelet, and cholesterol-lowering activities [30]. Studies have shown that vitamin E, specifically the tocotrienols was able to maintain bone density and prevent further bone loss in different animal models of osteoporosis [31]. Recent studies offered evidence for tocotrienols as a bone anabolic agent in normal male, ovariectomised female and nicotine-treated male rats [32–35]. Tocotrienols, similar to statins, suppress the activity of HMGCoA reductase (Figure 1), although through different mechanisms [36, 37]. Statins inhibit the enzyme activity through competitive inhibition, while tocotrienols modulate the intracellular mechanism of controlled degradation of the reductase protein [38, 39]. A prior study revealed that only gamma and delta tocotrienols stimulate the degradation of HMGCoA reductase, and only the delta isomer was able to block the cleavage of sterol regulatory element-binding proteins (SREBP) [39]. Therefore, administration of statins and delta-tocotrienol together may have synergistic or additive effects. Additionally, with coadministration of delta-tocotrienol, we may be able to avoid the occurrence of the adverse effects of high doses of lovastatin in humans.

The annatto bean is one of the major sources of tocotrienols, containing 90% delta and 10% gamma tocotrienols. The annatto tree is a tropical South American tree (*Bixa Orellana*), having spinose capsules with seeds and cordate leaves that yield annatto beans. A previous study reported that coadministration of a pure extract of annatto tocotrienols lowered the effective dose of lovastatin and offered a novel approach to cancer prevention and therapy [40]. Small daily doses of delta and gamma tocotrienols isolated from annatto bean reduced serum levels of cholesterol, triglycerides, and LDL by 15–20% [41]. Annatto-derived tocotrienol was chosen for this study due to the reported efficacy above, as well as the total absence of any tocopherol isomers in the extract. Tocopherol may interfere with tocotrienol absorption and distribution and may attenuate the inhibitory effect of delta-tocotrienol on liver HMGCoA reductase [42–44]. Previous studies have found that the tocopherol isomers do not prevent bone loss in orchidectomised rats [45, 46]. Thus, it is important to use a tocopherol free extract in this study.

Ovariectomised rats are a widely accepted model of postmenopausal osteoporosis due to their appropriateness, convenience, and relevance. Furthermore, the ovariectomised rats exhibit skeletal response similar to postmenopausal women [47].

Biochemical markers of bone resorption and formation are sensitive markers that reflect the different processes involved in bone metabolism by detecting the activity of osteoclasts and osteoblasts. However, they do not show the changes in bone mass and structure [48, 49]. Osteocalcin is an osteoblast-specific noncollagenous protein. It forms about 10% of noncollagenous proteins of the bone matrix and generally serves as a specific marker for osteoblast activity and bone formation [50]. Cross-linked C-terminal (CTX) telopeptides are proteolytic fragments of type 1 collagen formed during bone resorption. CTX is known as a specific marker for osteoclast activity and bone resorption [51]. Static bone histomorphometric indices are used to examine bone histology and quantitatively evaluate the activity of the bone cells at a specific time. Therefore, a strong tool to study bone metabolism and bone morphology is through a combination of bone biochemical analysis and static histomorphometric indices.

The current study was designed to evaluate the combined effects of delta-tocotrienol and lovastatin and to compare it with delta-tocotrienol and lovastatin given individually on bone biomarkers and static bone histomorphometric parameters in the ovariectomised estrogen-deficient female rat. The findings from this study may provide an alternative medication to treat postmenopausal osteoporosis.

## 2. Method and Materials

### 2.1. Animals

Forty eight female Sprague-Dawley rats that were approximately 3 months old and weighed 200–250 g, were purchased from the Laboratory Animal Research Unit, Universiti Kebangsaan Malaysia. The rats were kept two per cage under 12 hour light-dark cycles. The rats were fed commercial rat chow (Gold Coin, Selangor, Malaysia) and tap water *ad libitum*. After one week of acclimatization, the rats were randomly divided into 6 groups with 8 rats in each group. The first group, served as a baseline control (BC), was not ovariectomised and was sacrificed upon receipt. The second group was not ovariectomised but was sham-operated (SHAM) for simulation of surgical stress. The third group was the ovariectomised control group (OVXC). The fourth group was ovariectomised and treated with 11 mg/kg of lovastatin (OVX + LOV). The fifth was ovariectomised and treated with 60 mg/kg of delta-tocotrienol (OVX + TT). And the sixth was ovariectomised and treated with 11 mg/kg of lovastatin and 60 mg/kg of delta-tocotrienol (OVX + TT + LOV). The treatment had been administrated to the rats daily via oral gavage for 8 weeks.

Prior approval for the study protocol had been obtained from the UKM Animal Ethics Committee, (PP/FAR/2011/IMA/27-JANUARY/352-JANUARY-2011–DECEMBER-2012).

### 2.2. Preparation of Treatment

The Delta Gold 70 viscous oil (American River Nutrition, Hadely, USA) is a rich delta-tocotrienol extract from the annatto bean consisting of 90% delta-tocotrienol and 10% gamma-tocotrienol. The orange-red oil was diluted in olive oil (Bertolli Classico, Italy) and administrated daily via oral gavage at a dose of 60 mg/kg delta-tocotrienol for 8 weeks. This dose was roughly equivalent to 420 mg/day for an adult human.

Mevacor tablet, containing 40 mg of lovastatin, was crushed and suspended in 0.5% carboxymethylcellulose (Sigma-Aldrich, St. Louis, USA) solution and given daily to rats via oral gavage at a dose of 11 mg/kg for 8 weeks. This dose was equivalent to 80 mg/day for an adult human. Oral gavages of the vehicles were given to SHAM and OVX groups for a similar duration of treatment. The duration of the study was based on a previous study, in which 8 weeks was shown to be adequate for significant changes in bone parameters to be observed [52].

### 2.3. Sample Collection

For the biochemical study, blood samples were collected at the start (pretreatment) and after 8 weeks of treatment (posttreatment) from all the groups except BC because they were sacrificed upon receipt. Blood samples were obtained from the retroorbital vessel after the rat was anesthetized with diethyl ether. After 3 hours, blood was centrifuged for 10 min at 3000 rpm, and the serum stored at −70°C for further use.

For bone histomorphometric analysis, the rats were sacrificed by high dose diethyl ether after completing the treatment period. The left femurs were removed and the distal portion kept in 70% alcohol.

### 2.4. Biochemical Analysis

Levels of bone biochemical markers, osteocalcin and CTX in serum were measured using an ELISA microplate reader (VERSA max, Sunnyvale, USA). The kits used were Rat-Mid Osteocalcin ELISA kit (IDS, UK) and RatLaps CTX-1 ELISA kit (IDS, UK).

### 2.5. Bone Histomorphometry

The left femur was decalcified with EDTA (Sigma Aldrich, St. Louis, USA) for 2 months and then embedded in histological paraffin wax. The decalcified paraffin blocks were sectioned at 6 *μ*m with a microtome (Leica, Wetzlar, Germany) and stained with Hematoxylin and Eosin.

The static parameters, namely, osteoblast surface/bone surface (ObS/BS), osteoclast surface/bone surface (OcS/BS), eroded surface/bone surface (ES/BS), osteoid surface/bone surface (OS/BS), and osteoid volume/bone volume (OV/BV) were analysed using a quantitative stereological method for histology known as the Weibel technique.

The static histomorphometric indices were performed at the secondary spongiosa area, which is rich in trabecular bone. The selected metaphyseal region was located 1 mm from the lateral cortex and 3–7 mm from the lowest point of the growth plate.

Bone cellular average changes were analyzed and expressed using bone histomorphometric measurements as recommended by The American Society of Bone Mineral Research Histomorphometry Nomenclature Committee [53].

### 2.6. Statistical Analysis

Data analysis was performed using the Statistical Package for Social Sciences software (19, SPSS, Chicago, IL, USA). The Kolmgorov-Smirnov test was used as a normality test. The paired-sample *t* test was utilized to compare the same group before and after treatment. The ANOVA followed by post hoc Tukey's tests were used to determine the statistical significance between groups. The results were expressed as mean values ± standard error of the mean (SEM). The statistical differences were considered significant at *P* < 0.05.

## 3. Results

**Figure 2:**
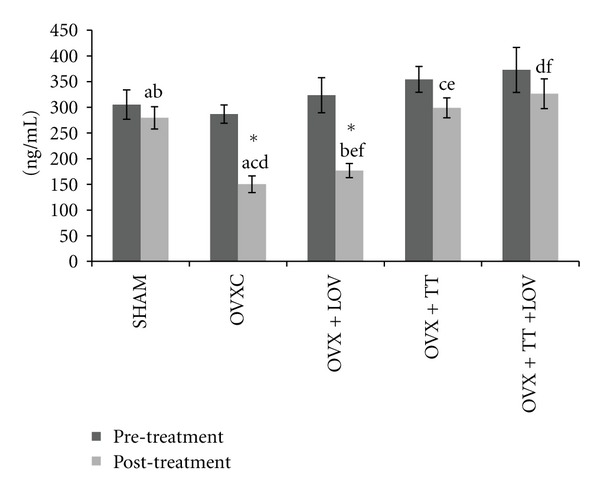
Serum osteocalcin levels in treatment groups. Data labeled with the same letter indicates significant difference between treatment groups. *Indicates significant difference between pretreatment and posttreatment values for the same group. Data was presented as mean ± SEM. Significant level was taken at *P* < 0.05.

Serum osteocalcin level was significantly lower post-treatment compared to pretreatment for the OVXC and OVX + LOV groups. The posttreatment level of serum osteocalcin did not differ significantly from the pre-treatment level for the remaining groups. No significant differences were seen between the groups before treatment. After treatment, the serum osteoclacin level in the OVXC group was significantly lower than the SHAM group. The OVX + TT and OVX + TT + LOV groups had significantly higher serum osteocalcin levels compared to the OVXC and OVX + LOV groups, but they did not differ from the SHAM group. While the OVX + LOV group did not differ significantly from the OVXC group but was significantly lower than the SHAM group (Figure 2).

**Figure 3:**
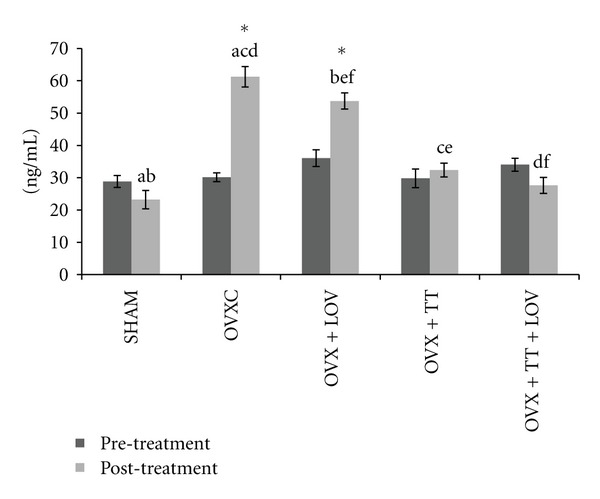
Serum CTX levels in treatment groups. Data labeled with the same letter indicates significant difference between treatment groups. *Indicates significant difference between pretreatment and posttreatment values for the same group. Data was presented as mean ± SEM. Significant level was taken at *P* < 0.05.

Serum CTX level was significantly higher posttreatment compared to pretreatment for the OVXC group. The posttreatment level of serum CTX did not differ significantly from the pretreatment level for the remaining groups No significant differences were observed between the groups before treatment. After treatment the serum CTX level for the OVXC group was significantly higher than the SHAM group. The OVX + TT and OVX + TT + LOV groups had significantly lower serum CTX levels compared to the OVXC and OVX + LOV groups, but they did not differ from the SHAM group. While the OVX + LOV group did not differ significantly from the OVXC group but was significantly higher than the SHAM group (Figure 3).

**Figure 4:**
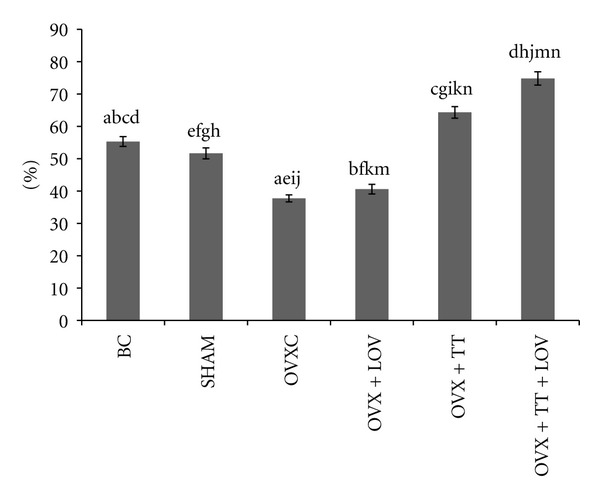
Osteoblast Surface/Bone Surface% (ObS/BS%) in treatment groups. Data labeled with the same letter indicates significant difference between treatment groups. Data was presented as mean ± SEM. Significant level was taken at *P* < 0.05.

**Figure 5:**
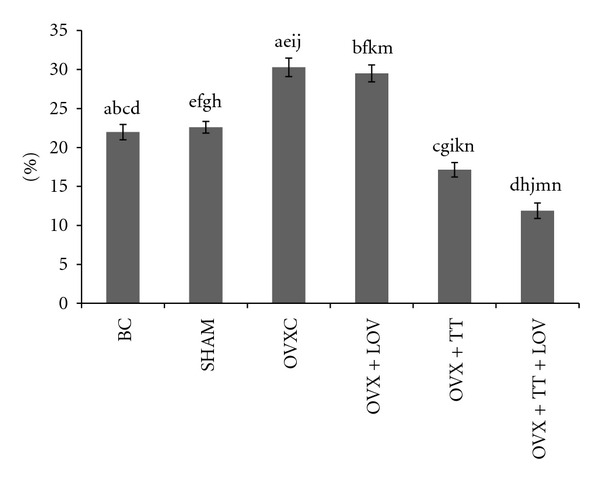
Osteoclast Surface/Bone Surface% (OcS/BS%) in treatment groups. Data labeled with the same letter indicates significant difference between treatment groups. Data was presented as mean ± SEM. Significant level was taken at *P* < 0.05.

**Figure 6:**
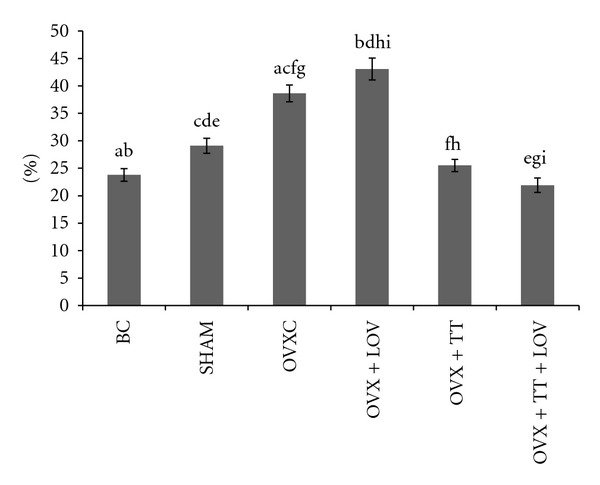
Eroded Surface/Bone Surface% (ES/BS%) in treatment groups. Data labeled with the same letter indicates significant difference between treatment groups. Data was presented as mean ± SEM. Significant level was taken at *P* < 0.05.

**Figure 7:**
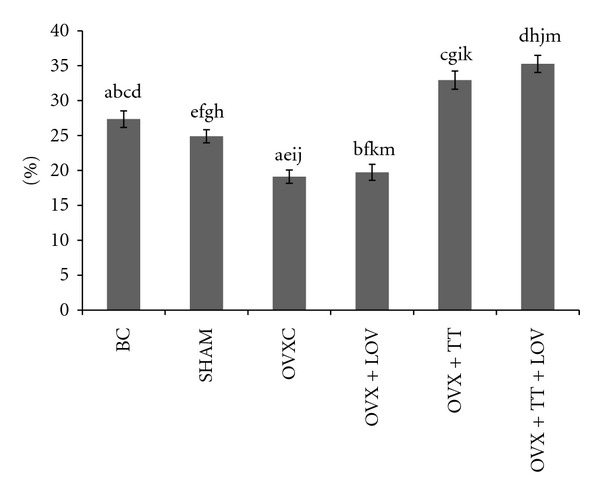
Osteoid Surface/Bone Surface% (OS/BS%) in treatment groups. Data labeled with the same letter indicates significant difference between treatment groups. Data was presented as mean ± SEM. Significant level was taken at *P* < 0.05.

**Figure 8:**
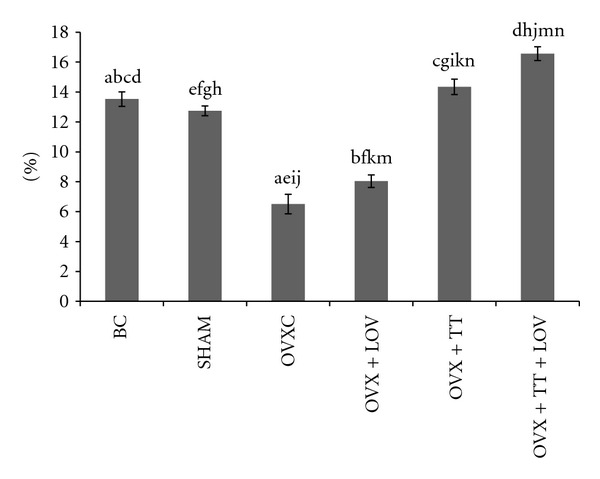
Osteoid Volume/Bone Volume% (OV/BV%) in treatment groups. Data labeled with the same letter indicates significant difference between treatment groups. Data was presented as mean ± SEM. Significant level was taken at *P* < 0.05.

The OVXC group had significantly lower ObS/BS, OS/BS and OV/BV values than the BC and SHAM groups (Figures 4, 5, 6, 7, and 8). There were no significant changes in all static bone parameters between the BC and SHAM groups. The OVX + TT + LOV group had significantly higher ObS/BS and OV/BV values compared to the OVX + TT group; significantly higher ObS/BS, OS/BS, and OV/BV values compared to OVX + LOV and OVXC groups; and significantly higher ObS/BS, OS/BS, and OV/BV values than the BC and SHAM groups. The OVX + TT group had significantly higher ObS/BS, OS/BS, and OV/BV values compared to the OVX + LOV and OVXC groups, and significantly higher ObS/BS, OS/BS, and OV/BV values than the BC and SHAM groups. The OVX + LOV did not differ from the OVXC in all static bone parameters but had significantly lower ObS/BS, OS/BS, and OV/BV values than the BC and SHAM groups (Figures 4, 5, 6, 7, and 8).

The OVXC group had significantly higher OcS/BS and ES/BS values than the BC and SHAM groups. The OVX + TT + LOV group had significantly lower OcS/BS value compared to the OVX + TT group; significantly lower OcS/BS and ES/BS values compared to the OVX + LOV and OVXC groups; significantly lower OcS/BS value than the BC and SHAM groups; significantly lower ES/BS value than the SHAM group. The OVX + TT group had significantly lower OcS/BS and ES/BS values compared to the OVX + LOV and OVXC groups; and significantly lower OcS/BS value than the BC and SHAM groups. The OVX + LOV group had significantly higher OcS/BS and ES/BS values than the BC and SHAM groups (Figures 4, 5, 6, 7, and 8).

## 4. Discussion

Both osteoblast and osteoclast cells are required for continuous bone remodeling. During bone formation, the osteoblast cells start to secrete osteoid and synthesize osteocalcin, while during bone resorption, the activated osteoclast cells dissolve the bone matrix resulting in the formation of the eroded surfaces and the release of CTX [54].

The results of the current study showed that daily supplementation of delta-tocotrienol in combination with lovastatin increased the osteoblastic bone formation and decreased osteoclastic bone resorption in ovariectomised rats as indicated by the OVX + TT + LOV group which had significantly higher serum osteocalcin, ObS/BS, OS/BS, and OV/BV values and significantly lower serum CTX, OcS/BS, and ES/BS values compared to the OVXC group. The role of the mevalonate pathway in the pathophysiology of osteoporosis suggests that critical regulatory mechanisms are needed to maintain osteoblast and osteoclast function. Inhibition of the mevalonate pathway by statins and tocotrienols (Figure 1) suppresses the prenylation of GTPase binding proteins and disrupts their function. Therefore, inhibition of GTPase function reduces the activity of osteoclasts and induces their apoptosis [5, 55–57]. Inhibition of GTPase function also increases osteoblast activity through enhancement of BMP-2 expression [5, 6, 57–60]. Ultimately, this will lead to stimulation of bone formation and decrease in bone resorption.

Competitive inhibition of HMGCoA reductase by statins reduces the cholesterol level. This reduction subsequently stimulates SREBP cleavage and inhibits HMGCoA reductase degradation, resulting in an increase in mRNA and HMGCoA reductase protein expression [61]. In contrast, delta-tocotrienol inhibits the cleavage of SREBP and induces the degradation of HMGCoA reductase, thereby inducing the reduction in mRNA and protein HMGCoA reductase levels [61]. Therefore, the combination of lovastatin and delta-tocotrienol may have synergistic or additive effects on bone metabolism, while at the same time avoiding the unwanted effects of high doses and low bioavailability of lovastatin.

The current study found that delta-tocotrienol combined with lovastatin provided better bone formation and bone protection against ovariectomy-induced bone loss compared to delta-tocotrienol alone as indicated by the OVX + TT + LOV group which had significantly higher ObS/BS, and OV/BV values and significantly lower OcS/BS value compared to the OVX + TT group. The improvement in bone metabolism by the combined treatment may be due to synergistic or additive inhibition of the mevalonate pathway. Moreover, the OVX + TT group had significantly higher serum osteocalcin, ObS/BS, OS/BS, and OV/BV values and significantly lower serum CTX, OcS/BS and ES/BS values compared to the OVXC group. These results were consistent with those who found that 60 mg/kg of tocotrienols had antiosteoporotic effects in thyroidectomised, orchidectomised, oxidative stressed, adrenalectomized, nicotine treated, and ovariectomised rat models [62–69]. The dose of 60 mg/kg/day for rats is roughly equivalent to 420 mg/day for humans, taking into the account the metabolic rate of rodents is around ten times faster than that of humans. This dose is relatively low has no toxic effects. It had been reported that daily supplementation of 200 mg/kg palm vitamin E extract containing 18.43% alpha-tocopherol, 14.62% alpha-tocotrienol, 32.45% gamma-tocotrienol, and 23.93% delta-tocotrienol has no toxic effects in female mice [70].

The current study showed that the combination of delta-tocotrienol plus lovastatin increased bone formation and reduced bone loss compared to lovastatin alone as indicated by the OVX + TT + LOV group which had significantly higher serum osteocalcin level, ObS/BS, OS/BS, and OV/BV values and significantly lower serum CTX, OcS/BS, and ES/BS values compared to the OVX + LOV group. Moreover, there were no significant changes in all biochemical markers and static bone histomorphometric indices between the OVXC and OVX + LOV groups. Therefore, lovastatin alone failed to enhance bone formation and to prevent bone resorption in ovariectomised rats at clinically tolerable hypocholesterolemic doses. Statins have limited distribution to the peripheral tissues after oral administration [71]. Therefore, they yield uncertain results as bone anabolic agents when used *in vivo* at cholesterol lowering doses. Bjarnason et al. [26] reported that fluvastatin did not affect serum osteocalcin and serum and urinary CTX levels in postmenoposal women with osteoporosis and mild hypercholesterolemia when given in clinically relevant doses. A cross over clinical study showed that 40 mg/day of atorvastatin had no effect on serum osteocalcin and CTX in type 2 diabetic men with baseline hypercholesterolemia compared to placebo [72]. Similar results were seen, when a randomized clinical trial measured the serum CTX concentration in hypercholesterolemic patients treated with 20–80 mg/day of simvastatin [24]. Twenty mg/day of pravastatin did not affect the serum CTX level in hypercholesterolemic postmenopausal women [25]. Meta analysis of both observational studies and clinical trials of around 300,000 patients found that there was clinical benefit from the use of oral statins but there was no significant reduction in fracture incidence in older women [73]. Yao and his coworkers ascertained that the 0.3, 0.6, 3, 6, and 10 mg/kg of simvastatin for 60 days could not prevent or restore ovariectomy-induced osteoporosis [74]. On the other hand, previous studies showed that lovastatin and other statins enhanced bone formation and reduced bone resorption after high oral doses in rodents [5, 7–10]. This indicates that clinically nontolerable doses of oral statins are required to achieve successful prevention and treatment of osteoporosis. Myotoxicity and hepatotoxicity were associated with the high doses of oral statins [27–29]. In this study, 11 mg/kg of lovastatin was chosen, which if extrapolated to human is roughly equivalent to 80 mg/day, the highest dose of lovastatin used as an antihyperlipidemic agent.

The results of the current study found that the OVX + TT + LOV group had significantly higher ObS/BS, OV/BV and OS/BS values and significantly lower OcS/BS and ES/BS values than the SHAM group. These current findings indicate that delta-tocotrienol in combination with lovastatin promoted better cellular bone histomorphometric parameters than the SHAM group, thus exhibiting bone anabolic effects. Therefore, the combined treatment has the potential to increase bone strength. Recently, tocotrienols were shown to have bone anabolic activity in ovariectomised female, intact male and nicotine-treated male rats [32–35], and these findings had been confirmed by the results of the current study (Figures 4, 5, 7, and 8). Therefore, combination of delta-tocotrienol plus lovastatin may have the ability to further improve the bone density in normal bone.

## 5. Conclusion

Supplementation of delta-tocotrienol in combination with oral statins at clinically acceptable doses has both bone antiosteoporotic and anabolic activity and was more effective than delta-tocotrienol and lovastatin given individually. Therefore, the combination of delta-tocotrienol plus lovastatin has the potential to be used as an anti-osteoporotic agent especially in patients who are at risk of both conditions, that is, osteoporosis and hypercholesterolemia. This is especially true for postmeanopausal women, and also for men of the older age group.
